# Multidetector computer tomography in the pancreatic adenocarcinoma assessment: an update

**DOI:** 10.1186/s13027-016-0105-6

**Published:** 2016-11-15

**Authors:** Vincenza Granata, Roberta Fusco, Orlando Catalano, Sergio Venanzio Setola, Elisabetta de Lutio di Castelguidone, Mauro Piccirillo, Raffaele Palaia, Roberto Grassi, Francesco Granata, Francesco Izzo, Antonella Petrillo

**Affiliations:** 1 Department of Diagnostic Imaging, radiant and metabolic Therapy, Istituto Nazionale Tumori IRCCS Fondazione Pascale, Naples, Italy; 2 Department of Hepato-Biliary Surgery, Istituto Nazionale Tumori IRCCS Fondazione Pascale, Naples, Italy; 3 Departement of Radiology, Seconda Università degli Studi di Napoli, Naples, Italy; 4 Departement of Civil and Mechanical Engineering, University of Cassino and Southern Lazio, Lazio, Italy

**Keywords:** Pancreatic adenocarcinoma, Multidetector computer tomography, Perfusion CT, Dual-source CT

## Abstract

Ductal adenocarcinoma of the pancreas is one of the most aggressive forms of cancer, with only a minority of cases being resectable at the moment of their diagnosis. The accurate detection and characterization of pancreatic carcinoma is very important for patient management. Multidetector-row computed tomography (MDCT) has become the cross-sectional modality of choice in the diagnosis, staging, treatment planning, and follow-up of patients with pancreatic tumors. However, approximately 11% of ductal adenocarcinomas still remain undetected at MDCT because of the lack of attenuation gradient between the lesion and the adjacent pancreatic parenchyma. In this systematic literature review we investigate the current evolution of the CT technique, limitations, and perspectives in the evaluation of pancreatic carcinoma.

## Background

Pancreatic adenocarcinoma is one of the most aggressive forms of cancer. It is the fourth most common cause of cancer-related mortality worldwide. The prognosis of pancreatic cancer is still bleak, as the 5-year survival rate is less than 5% and the mortality rate has not declined over the last few decades, with an increasing global incidence of nearly 340,000 in 2012. The incidence rate near equaling that of its mortality rate [1]. At presentation, about 40% of patients with pancreatic cancer are diagnosed with metastatic disease (stage IV) and 40% are diagnosed with locally-advanced pancreatic cancer (LAPC) [1, 2].

The accurate characterization of pancreatic adenocarcinoma is very important for patient management. Computer tomography (CT) and magnetic resonance imaging (MRI) are the most important modalities for evaluating pancreatic lesions. A precise diagnosis of pancreatic tumor is not always simple because the tumor can have atypical imaging features and many other disorders may mimic pancreatic adenocarcinoma [3, 4]. Multidetector row (MD) CT has become the modality of choice in the preoperative diagnosis and staging of the disease and in treatment planning and follow-up in patients with pancreatic tumors [5]. Although many studies have investigated the effect of different techniques of contrast medium injection for improving the enhancement of the pancreas and peripancreatic vasculature during the pancreatic parenchymal phase [6–10], approximately 11% of ductal adenocarcinomas remain undetected at MDCT. This is because of the lack of a visible attenuation difference between the tumor and the adjacent pancreatic parenchyma. In these cases, recognition secondary signs (e.g. main pancreatic duct dilatation or the interrupted duct sign) becomes mandatory to detect the lesion [11]. In this review we investigate the evolution of the MDCT technique, limitations, and future prospects in the evaluation of pancreatic adenocarcinoma.

## Materials and Methods

Data for this review were identified by searches of the PubMed database using a multimodal strategy. The following search terms were employed: CT in pancreatic cancer, functional CT in pancreatic cancer, CT in advanced pancreatic cancer, CT in advanced pancreatic adenocarcinoma after chemotherapy. The inclusion criteria were: clinical study evaluating pancreatic adenocarcinoma, clinical study evaluating new functional imaging criteria in the CT study of patients with pancreatic adenocarcinoma, and clinical study evaluating follow-up after chemotherapy of patients with advanced pancreatic adenocarcinoma. Articles published in the English language from January 2003 to June 2016 were included. The references of these articles were also analyzed to identify original studies that were not identified by the search of the data. Exclusion criteria unavailability of full text and absence of original research data (reviews, editorials, case reports, etc.).

## Results

**Fig. 1:**
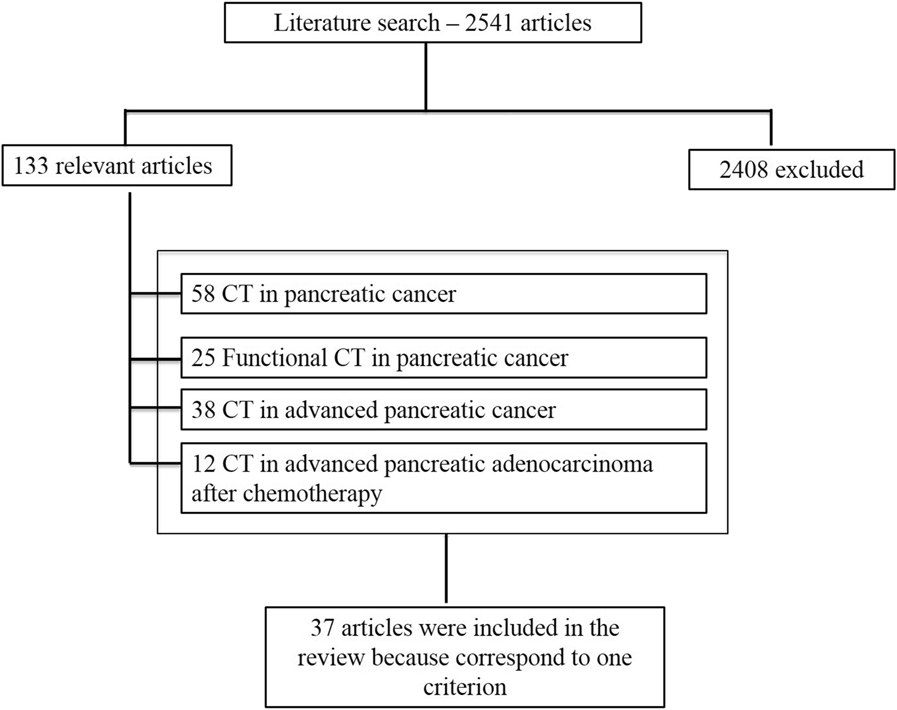
Included and excluded studies in systematic review

A Pubmed search yielded 1821 articles for key CT in pancreatic cancer, 129 articles for functional CT in pancreatic cancer, 514 articles for CT in advanced pancreatic cancer, 77 articles for CT in advanced pancreatic adenocarcinoma after chemotherapy. Among the total number of 2541 articles, 2408 were excluded because of unmatching the inclusion criteria. In the end, there were 133 articles: 58 for CT in pancreatic cancer, 25 for functional CT in pancreatic cancer, 38 for CT in advanced pancreatic cancer, and 12 for CT in advanced pancreatic adenocarcinoma after chemotherapy. Among these, 96 articles corresponded to more than one criterion so 37 articles were included at the end (Fig. 1).

## Discussion

**Fig. 2:**
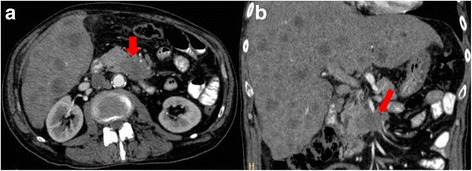
CT scan in axial plane (**a**) and coronal plane, Multiplanar Reconstruction-MPR (**b**), during pancreatic phase of dynamic contrast study. Body-tail adenocarcinoma (arrow)

**Fig. 3:**
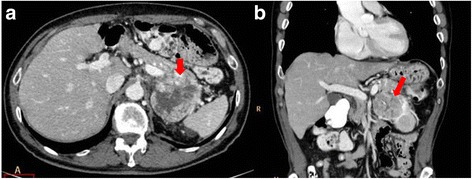
CT scan in axial plane (**a**) and coronal plane, MPR (**b**), during portal phase of dynamic contrast study. Tail pancreatic adenocarcinoma that infiltrates vascular hilum of the spleen

**Fig. 4:**
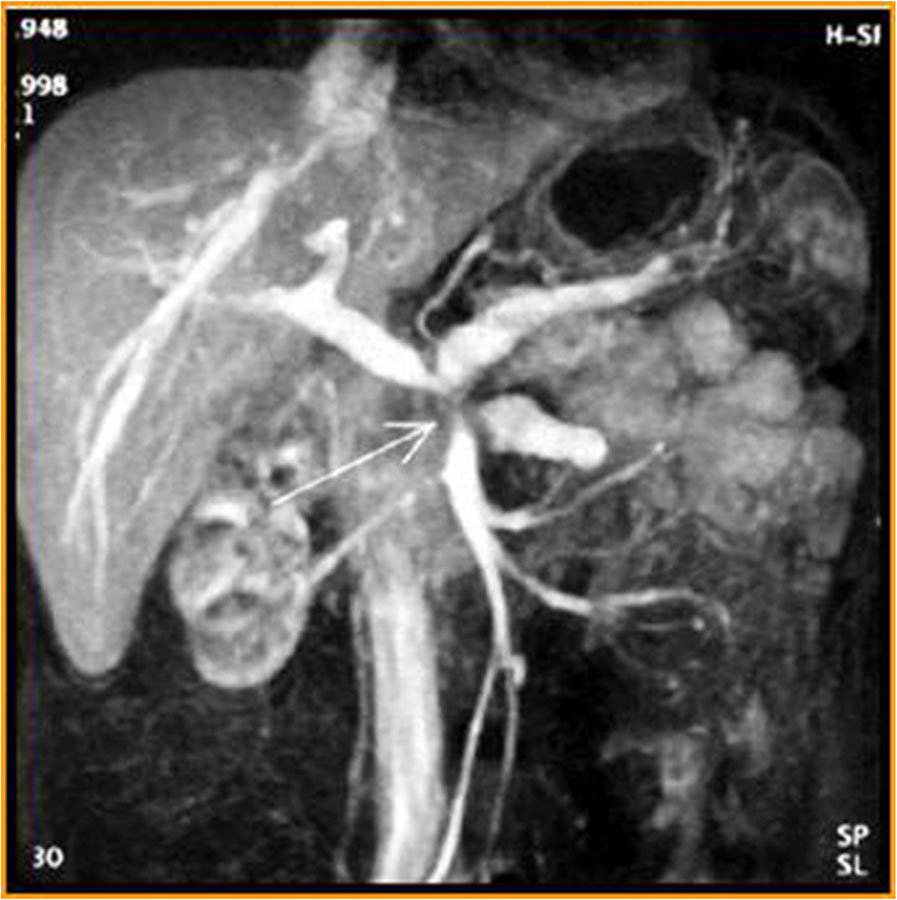
Maximum Intensity Projection (MIP); arrow shows vascular infiltration

MDCT has become the modality of choice in the preoperative diagnosis and staging and in treatment planning and follow-up of patients with pancreatic tumors [5]. Since its introduction in clinical practice, volumetric CT scanning has revolutionized pancreatic imaging, although the greatest benefits have occurred with the development of MDCT. This basically included short acquisition times, thin sections in a single-breath hold, retrospective calculation of thinner or thicker sections from the same raw data, and improved 3D post-processing [12]. The detection of tumor tissue and visualization of pancreas and vessel infiltration is needed so as to define when a tumor is resectable [12]. To visualize organ infiltration (Figs. 2 and 3) a good contrast-to-noise ratio is mandatory. To diagnose vessel infiltration, the optimal imaging phase must be timed (Fig. 4) [13].

The enhancement kinetics of the normal pancreas follows arterial dynamics. There is a longer delay necessary to increase the enhancement of the interstitial spaces of the organ of interest, and hence to increase the contrast to the ductal system and the lesion-to-background contrast for hypovascular pancreatic adenocarcinomas. Pancreatic imaging thus also benefits from high iodine flux and accurate scan timing relative to the arrival of contrast media in the abdominal aorta but also improves with larger contrast medium volumes. Scan timing is critical, and best lesion-to-background contrast is achieved approximately 20 to 25 s after contrast medium arrival in the aorta (for injection durations of 30 s, and scan times of approximately 5 s) [13].

**Fig. 5:**
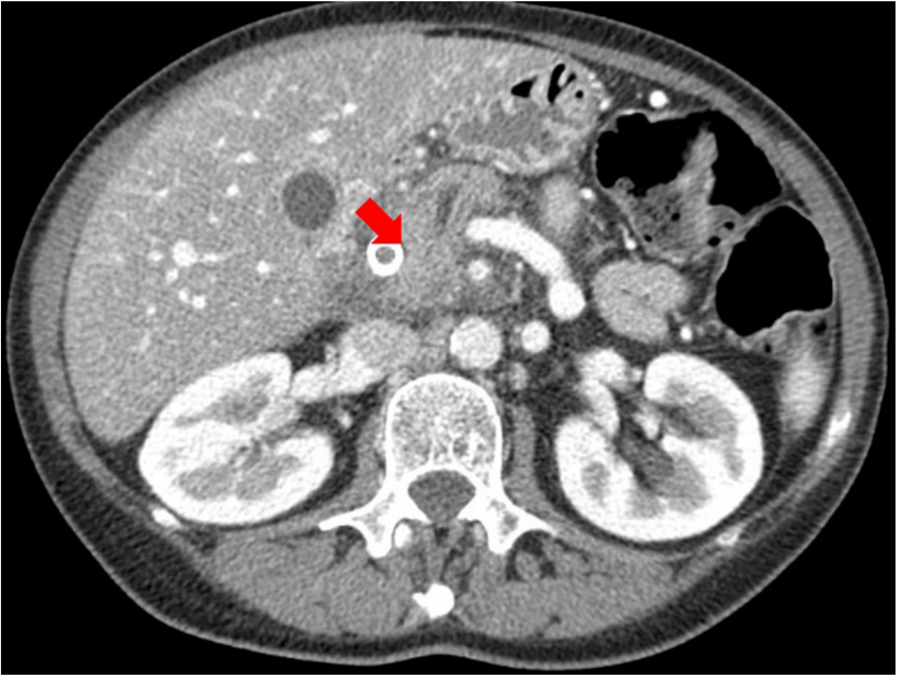
CT scan in axial plane during pancreatic phase of dynamic contrast study. Isodense pancreatic adenocarcinoma (arrow)

Pancreatic parenchyma study should be carried out with a quadruple-phase scanning protocol, including unenhanced, arterial, pancreatic parenchymal, and portal venous phases. However, according to Fletcher [14], the acquisition of images in the arterial phase is not necessary in the detection and staging of pancreatic adenocarcinoma. The acquisition of images in the pancreatic phase is adequate to detect pancreatic tumor because in this phase tumor-to-gland attenuation differences are greatest. The images obtained during the hepatic phase allow a tumor detection rate nearly equivalent to that of the images obtained during the pancreatic phase [14]. Additionally, hepatic-phase images most accurately depict vascular invasion [14]. So, to guarantee the most effective rate of lesion detection, CT images must be acquired during the pancreatic parenchymal phase when the maximum difference in attenuation is attained between typically poorly vascularized pancreatic tumors and vividly enhancing pancreatic parenchyma [14]. Current MDCT protocols maximize the attenuation differences between the hypovascular tumor and the surrounding parenchyma [15]. However, there is an 11% of pancreatic adenocarcinoma being isoattenuating (Fig. 5) to the pancreatic parenchyma [16]. Consequently, many studies have investigated the effect of techniques for injection of contrast media on improving the enhancement of the pancreas and peripancreatic vasculature during the pancreatic parenchymal phase [6–10]. To improve the conspicuity of pancreatic tumor and reduce radiation dose, Marin et al. [10] evaluated the so called the low-tube-voltage, high-tube-current CT technique. These researchers demonstrated that, compared with a high-tube-voltage CT protocol (140 kVp), a low-tube-voltage (80 kVp), high-tube current (675 mA) technique has the potential to improve the enhancement of the pancreas and peripancreatic vasculature, increasing tumor conspicuity and reducing patient radiation dose during the pancreatic parenchymal phase. Nonetheless, these improvements come at the cost of decreased perceived image quality (primarily the result of increased noise) beyond what most clinical radiologists would consider acceptable [10]. Dual-energy CT techniques may be used to distinguish substances such as iodine, calcium and uric acid crystals from soft tissues. The use of dual-energy CT has potential clinical implications for imaging of the pancreas [10, 17–25]. Owing to increased photoelectric absorption and less Compton scatter at the lower photon energies, the attenuation of contrast material is greater at 80 kVp than at 120 or 140 kVp. The closer the energy level is to the K edge of a substance such as iodine, the more the substance attenuates. The term K edge refers to the spike in attenuation that occurs at energy levels just greater than that of the K-shell binding because of the increased photoelectric absorption at these energy levels. K edge values vary for each element, increasing as the atomic number increases. With current dual-energy CT technology, the two energies most frequently employed are 80 kVp and 140 kVp. Because the K edge of iodine (33.2 keV) is closer to 80 kVp than it is to 140 kVp, the attenuation of iodine-containing substances is substantially higher at 80 kVp [17]. According to Patel et al. [18] Single-source, dual-energy MDCT could offer better ability to detect hypovascular pancreatic adenocarcinomas at lower viewing energy levels during the pancreatic phase of imaging. Consequently the number of “under detected”, early-stage lesions or isoattenuating tumors would decrease. Macari et al. evaluate the attenuation and conspicuity of pancreatic neoplasms and the pancreatic duct at dual-energy dual-source CT performed at 80 kVp compared with the more typical 120 kVp. The study showed that in comparison with a weighted-average 120-kVp data set, generation of pure 80-kVp data improves differentiation of attenuation values between malignant tumors of the pancreas and normal pancreas and may increase the conspicuity of nearly isoattenuating and small pancreatic adenocarcinomas [19]. Zamboni et al. [20] tested a single-energy low-voltage CT protocol for pancreatic adenocarcinoma to compare an 80 kV arterial phase scan protocol with a 120 kV protocol, with regard to lesion conspicuity, image quality and radiation dose, both on a patient population and on phantoms. They demonstrated that the use of a low voltage technique for pancreatic phase scanning can increase even more the conspicuity of the tumor in significant dose reduction while maintaining an acceptable image quality [10, 17–25].

**Fig. 6:**
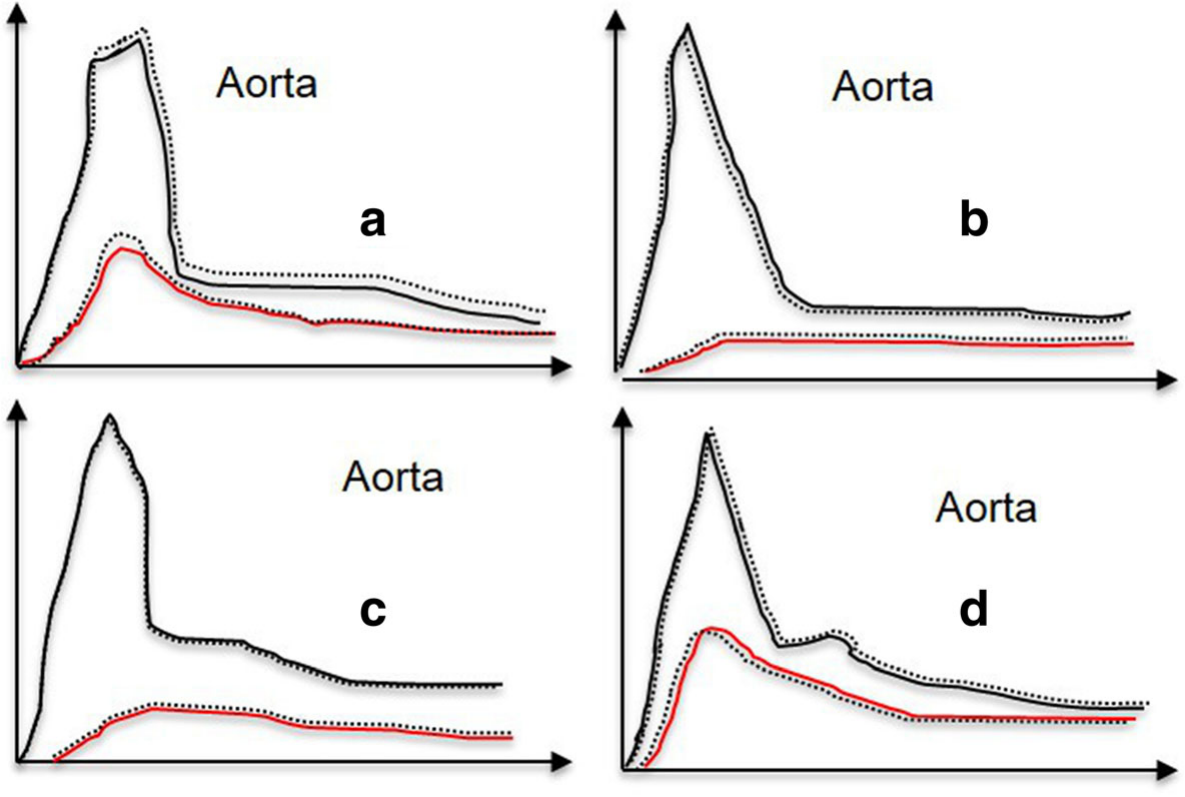
**a** Type A curve wash-in followed by wash-out (normal parenchyma); **b** type B curve-low wash-in, followed by plateau or increasing density, without wash-out (adenocarcinoma); **c** type C curve-low wash-in, followed by at least a slight wash-out (chronic pancreatitis); **d** type D curve-brisk wash-in, followed by clear wash-out (endocrine tumor)

Perfusion CT (CTp) is a clinical technique that can be used to provide maps and obtain quantitative measurements of various hemodynamic parameters on the basis of the linear relationship between CT enhancement and iodinated contrast material concentration [26–33]. Some study evaluated perfusion CT parameters, such as the volume transfer constant (Ktrans) between blood plasma and extracellular extravascular space and the blood volume calculated from dynamic CT data, or median peak enhancement intensity and median blood volume can be used to identify, to characterize and to evaluate the response of pancreatic cancer to chemo-radiotherapy [26–33]. Scialpi et al. [26] evaluated the effectiveness of CTp imaging to detect small (≤2 cm) pancreatic adenocarcinoma. These authors showed that the quantitative analysis of the enhancement for pancreatic adenocarcinoma and surrounding parenchyma may be considered in order to increase the sensitivity of CT in the detection of small tumor. Zamboni et al. [27] evaluated the rule of time-density curve morphology for the differential diagnosis of solid pancreatic lesions showed that morphology of the time-density curves obtained from serial scans after contrast medium injection could be used to aid in the differential diagnosis between normal parenchyma, tumors and atrophic parenchyma. Curve morphology B (Fig. 6), with no washout in the last portion of the curve corresponds to adenocarcinoma, while curve morphology C (Fig. 6), with at least some washout in the last portion of the curve, corresponds to chronic pancreatitis. Yamada et al. [28] reported the use of time-density curves obtained from triphasic CT in the differentiation between carcinoma and mass-forming chronic pancreatitis. They concluded that pancreatic adenocarcinoma shows an increasing contrast enhancement pattern, while chronic pancreatitis shows an early-washout pattern. Delrue et al. demonstrated the feasibility of CTp in patients with a pancreatic pathologies and showed that CTp is more capable to differentiate the pancreatic disorders, compared to density measurements alone, since no significant differences in perfusion values were found between acute/chronic pancreatitis and pancreatic adenocarcinoma, so differential diagnosis based only on CTp data remains difficult [29]. Functional imaging from CTp may add useful information on tumor aggressiveness affecting treatment strategy and patient management. D’Onofrio et al. evaluated the CTp features of locally advanced pancreatic ductal adenocarcinomas and assessed whether these features correlate with the tumor grading at pathology, showed that CTp can predict tumor grade of pancreatic adenocarcinoma. In particular, median peak enhancement intensity and median blood volume proved their efficiency in identifying high grade pancreatic adenocarcinoma [30]. Nishikawa et al. [31] investigate the relationship between prognosis and perfusion in the tissue surrounding cancer. This study proven a significant correlation between area under the curve of peritumoral tissue (AUCPTT) or blood flow of peritumoral tissue (BFPTT) and survival days from the date on which perfusion CT was performed: higher AUCPTT or BFPTT values were associated with shorter survival. While it has not been demonstrated a significant correlation between the BF of tumor or AUC of tumor with patient survival. These findings suggest that prognosis is related to increased perfusion in tissue surrounding cancer [31]. Park et al. [32] reported that decreased tumor permeability measured by CTp is related to chemosensitivity: pancreatic tumors with high pretreatment Ktrans values indicating higher intratumoral flow tended to respond better to the neoadjuvant chemotherapy. So, CTp may be used to predict the tumor response of neoadjuvant chemotherapy and radiation therapy in patients with pancreatic cancer [32]. CTp data can not only be markers which allow to discriminate responders from non-responders patients in the course of chemotherapy, but they can also be helpful in the prediction of potential postoperative complications: Sugimoto et al. [33] evaluated risk assessment for postoperative pancreatic fistula after pancreaticoduodenectomy using perfusion CT and showed that patients who developed fistulas showed a higher arterial flow and shorter mean transit time in the CTp profile, that reflected a lower main pancreatic duct ratio, lower fat ratio, lower fibrosis ratio, higher lobular ratio, and lower vessel density in the histological assessment of the pancreatic stump. Therefore, CTp may enable preoperative, objective, and quantitative assessment of the risk of complications and allow surgeons to choose appropriate countermeasures against postoperative pancreatic fistula [33]. Klauß et al. [34] evaluated the feasibility of dual-energy CT (DECT)-perfusion of pancreatic carcinomas for assessing the differences in perfusion, permeability and blood volume of healthy pancreatic tissue and histopathologically confirmed solid pancreatic carcinoma. The study showed that perfusion, permeability and blood volume values were significantly lower in pancreatic carcinomas compared to healthy pancreatic tissue; so the use of DECT improves the accuracy of CTp of the pancreas by fully exploiting the advantages of enhanced iodine contrast at 80 kVp in combination with the noise reduction at 140 kVp. Therefore using dual-energy perfusion data could improve the delineation of pancreatic carcinomas [34].

To reduce radiation dose and increase conspicuity of tumor Brook et al. [35] evaluated the Split-bolus technique during the study of the pancreas. Split-bolus contrast material administration is based on injection of various amounts of contrast material in two or three parts with a variable pause while scanning is performed only once, so to obtain a combined-phase images in a single scan. In this study has been demonstrated that with a single combined phase, split-bolus spectral CT examination resulted in vascular, liver, and pancreatic attenuation and pancreatic tumor conspicuity equal to or higher than those obtained with a standard combination of two-phase pancreatic CT, with a 43% reduction in the radiation dose [35].

To identify and characterize a pancreatic lesion is essential not only the phase of study but also the concentration, the injection flow rates and volumes. Yanaga et al. [36] evaluated a protocol with a fixed contrast material injection dose and one with a dose tailored to patient body weight and has been demonstrated that with the fixed contrast material dose protocol, pancreatic enhancement during the pancreatic parenchymal phase was significantly worse in the heavier patients (body weight of 60 kg or greater) than in the lighter patients (body weight of less than 60 kg). On the other hand, there was no significant difference between the two weight groups with the protocol in which contrast material dose was tailored to patient weight. So CT protocols that deliver a contrast material dose tailored to patient weight at a fixed injection duration yield satisfactory pancreatic enhancement in patients of different body weights [36] and that increases the tumor detection. Liu et al. [37] demonstrated that although the pancreas, pancreatic malignancies and other abdominal organs are more enhanced in higher iodine concentration group in arterial and/or portal venous phase, the detection and demarcation of pancreatic adenocarcinomas was not found to improve with the higher iodine concentration so as demonstrated also by Fenchel et al. [38]. These results demonstrate that where it has to use contrast agents at low dose, as in patients with renal impairment, there is not the risk of identifying a pancreatic tumor [37, 38]. Then for an accurate study of the pancreatic parenchyma it is more important than a correct dose of contrast, considering the patient’s body weight, which is not the concentration of the contrast [36–38].

Although radical resection is still the only curative treatment for pancreatic cancer, it is generally accepted that a multimodality strategy is necessary for its management, because at the time of diagnosis about 40% of patients with pancreatic cancer are diagnosed with metastatic disease (stage IV) and the remaining 40% are diagnosed with LAPC [1, 2, 39]. After neoadjuvant chemo-radiotherapy (CRT) or ablation therapy, CT is usually used to evaluate treatment response and to restage tumors. CRT, especially radiation therapy, may induce fibrosis, which confounds viable tumor analysis at CT reducing preoperative staging accuracy [40]. Also the study of Cassinotto et al. [41] confirmed the decrease in the diagnostic performance of the CT to reevaluate resectability after neoadjuvant therapy of pancreatic tumor. Morgan [42] demonstrated that the CT sensitivity for prediction of resectability tends to be lower for patients with locally advanced pancreatic cancer that has been downstaged by neoadjuvant therapy than for controls who did not receive preoperative therapy. In patients undergoing chemo radiotherapy the most limits of CT is differentiating a residual tumor from the fibro-inflammatory tissue, so other techniques are needed to optimally evaluate the tumor response. MRI with diffusion sequencing and dynamic analysis would enable to differentiate between tumor tissue and edematous fibrosis [2, 4, 39]. Similarly, CTp could help provide functional information and aid in the characterization of residual tissue [32].

## Conclusion

Although MDCT is the modality of choice in the preoperative diagnosis and staging and in treatment planning and follow-up of patients with pancreatic cancer, there is an 11% of pancreatic adenocarcinoma is being unrecognized. Therefore it is necessary to optimize the technique, with a dose of the contrast medium adequate to the weight of the patient, with a proper delay after administration of the contrast medium. Parenchymal pancreatic phase is better than arterial phase. Dual-source, dual-energy MDCT and CTp improve the detection of pancreatic adenocarcinoma, increasing the conspicuity of nearly isoattenuating, small lesions. CTp, additionally, provides functional information and can aid in the assessment of residual viable tumor tissue after CRT.

## Abbreviations

AUCPTT: Area under the curve of peritumoral Tissue
BFPTT: Blood flow of peritumoral tissue
CRT: Chemo-radiotherapy
CTp: Perfusion CT
DECT: Dual-energy CT
LAPC: Locally-advanced pancreatic cancer
MDCT: Multidetector-row computed tomography
MRI: Magnetic resonance imaging
